# Prevalence and relevant factors of nocturia and its impact on sleep quality in Chinese university students

**DOI:** 10.1038/s41598-024-60656-9

**Published:** 2024-06-16

**Authors:** Yakai Liu, Zhenwei Zhang, Huijie Hu, Xiangfei He, Pengchao Xu, Qifeng Dou, Cuiping Song, Huiqing Zhang, Israel Franco, Konstantinos Kamperis, Søren Rittig, Jianguo Wen

**Affiliations:** 1 Department of Pediatric Surgery, The First Affiliated Hospital of Xinxiang Medical University, Xinxiang, China; 2 Third Affiliated Hospital of Xinxiang Medical University, Xinxiang, China; 3 School of Nursing, Sanquan College of Xinxiang Medical University, Xinxiang, China; 4 Pediatric Urodynamic Center and Department of Urology, The First Affiliated Hospital of Zhengzhou University, Zhengzhou, China; 5 Department of Urology, the First Affiliated Hospital of Xinxiang Medical University, Xinxiang, China; 6 Yale New Haven Children’s Hospital, New Haven, CT USA; 7 Department of Child and Adolescent Health, Aarhus University Hospital, Palle Juul-Jensens Blvd. 99, 8200 Aarhus, Denmark; 8 Henan Joint International Pediatric Urodynamic Laboratory, First Affiliated Hospital of Zhengzhou University, Zhengzhou, 450000 China

**Keywords:** Nocturia, Prevalence, University students, Relevant factors, Sleep quality, Medical research, Risk factors, Signs and symptoms, Urology

## Abstract

The purpose of this study was to investigate the prevalence and relevant factors of nocturia and its impact on sleep quality in university students in Mainland China. A large-scale survey was conducted on 14,000 university students from 3 universities in Henan province, China by using an anonymous questionnaire. The questionnaire collected the information from the past six months. The relationships between the prevalence of nocturia and its relevant factors were evaluated. A total of 13,874 questionnaires were collected and 13,104 qualified for statistical analysis. A total of 659 students suffered from clinically relevant nocturia (CRN) (4.56% in male and 5.34% in female). Both univariate analysis and the logistic stepwise regression model showed that the prevalence of nocturia was significantly related to female, history of enuresis, ease of waking up, urgency, frequency and RUTI (*P* < 0.05). The sleep quality and the university entrance score of CRN group was significantly lower than that of control group (*P* < 0.05). Nocturia was common in Chinese university students and showed a negative impact on sleep and academic performance. Gender of female, history of enuresis, ease of waking up, urgency, frequency and RUTI were relevant factors for CRN.

## Introduction

In 2019, the International Continence Society (ICS) updated the definition for nocturia based on the number of times urine was passed during the main sleep period where an individual waked to pass urine and then sleeps or had the intention to sleep. The main sleep period was defined as the period running from the point at which one fell asleep to the time one intends to wake the following day. This definition included people who, due to their work or lifestyle habits (e.g., police officers and doctors who work all night), had very different sleep schedules than most people^1^. The new definition was more accurate than the old definition. Several studies had demonstrated that nocturia had negative impact on quality of life and general health^2–4^, including reduced sleep quality, mood disturbance, diurnal fatigue, and lower work performance. Unfortunately, nocturia was often unreported, with the general lack of knowledge that nocturia was a treatable medical condition being cited as a crucial barrier to seeking treatment.

Nocturia was very common among all age groups. Bosch and Weiss reviewed the nocturia prevalence studies in community based populations, predominantly in the western countries and some Asian countries (including Taiwan, Japan, Korea, Hong Kong and Singapore)^5^. They reported that prevalence of nocturia among younger populations (20–40 years old) with one or more voids per night varies from 11–44% whilst those with two or more voids per night had been reported to range between 2 and 18%. Similarly, an epidemiological survey of nocturia from Chinese mainland found that 57.5% of participants reported voiding once per night, and 24.7% of participants reported voiding twice or more per night, and the prevalence of nocturia increases with time and age^6^. However, in these studies, the average age of the studied patients was over 30 years old. The prevalence of nocturia in young university students and whether their sleep quality had the same effect remains unclear.

It was well know that nocturia was a highly prevalent disorder that negatively affects sleep, increases daytime tiredness, and adversely impacts quality of life^7,8^. The literature on the effect of nocturia on sleep quality in Chinese university students had not been reported. Understanding the prevalence of nocturia, its relevant factors, and its effects on the sleep quality could help define treatment strategies and develop preventive measures in the future.

Consequently, the purpose of this study was to investigate the prevalence and relevant factors of nocturia as well as its impact on the sleep quality of university students in Mainland China.

## Materials and methods

### Study participants

From September 2023 to Octomber 2023 a total of 14,000 young university students (17–23 years old) from 3 universities in Henan Province, China was surveyed. The students were all Chinese and came from 363 cities within 30 provinces of Mainland China. They all lived within the university dormitories, which in China, all had similar living environments with strict rules and regulations related to sleep times. In China, both high middle school and university had a rule of a regular sleep schedule, all need to go bed before 10:00PM in the evening and get up at 06:00AM in the morning. The subjects were fully informed of the content and information of the survey. Subjects provided written informed consent during the process of survey completion. Research had demonstrated that increased bother was associated with at least two voids per night^9,10^. Therefore, the study defined clinically relevant nocturia (CRN) as waking to urinate ≥ 2 times per night.

The survey was in the form of an anonymous questionnaire which was distributed by over 50 professionally trained investigators who personally met with the students in groups to explain the purpose and significance of the survey to ensure a high response rate. The questionnaire was pre-tested with a pilot study (n = 40, respectively) to assess the comprehension of the questions. Feedback was taken into consideration and the questionnaires were appropriately amended. A second pilot study was conducted after the changes made to the questions, followed by a retest of the pilot study to assess internal consistency. The students were also given an instruction pamphlet with further information to explain certain terms used to ensure accuracy and help ease the completion of the questionnaire. After filling out the questionnaire, the students were asked to place it in a sealed questionnaire box to maximize the privacy of the students. The relevant questions in the questionnaire have been verified in previous studies^11^

The study followed the principles expressed in the World Medical Association Declaration of Helsinki and the International Ethical Guidelines for Biomedical Research Involving Subjects (GIOMS, Geneva, 1993) and Chinese clinical research management regulations. The study program was approved by the medical ethics committee of the First Affiliated Hospital of Xinxiang Medical University (EC-023-471). Patient consent was obtained for data collection and analysis.

### Content of the questionnaire

The self-administered questionnaire included two sections requiring the participant to tick boxes. The first section documented demographic information, including the students’ date of birth, gender, university entrance score and inhabitation (i.e. living in a rural or urban area) of the student.

The second part included questions about the details of nocturia and relevant factors, including: (1) history of primary nocturnal enuresis (PNE; intermittent incontinence while asleep in a child > 5 years of age, at least twice a month^12^; (2) ease of waking at night; (3) previous treatment; (4) whether the respondent experienced frequency, urgency, or daytime urinary incontinence (DUI) during the daytime; and(5) whether a recurrent urinary tract infection (RUTI). RUTI was defined as two episodes of acute bacterial cystitis, along with associated symptoms within the last six months or three episodes within the last year^13^.

The PSQI questionnaire was used to evaluate sleep quality and disturbance over the past month^14^, with a higher score indicating poorer sleep quality. It contained 19 items. The first four items were open questions (For example: What time do you usually go to bed? How long will it take you to fall asleep? What time do you usually get up in the morning? How long do you actually sleep at night?), and items 5–19 were rated on a four-point scale. The item scores yield the following seven subscores ranging from 0 to 3: C1 (sleep quality), C2 (sleep latency), C3 (sleep duration), C4 (sleep efficiency), C5 (sleep disturbance), C6 (sleep medication use), and C7 (daytime dysfunction due to sleepiness). Total scores ranging from 0 to 21 were obtained by summing the seven sub scores.

### Exclusion criteria

For this study, only the Chinese students of 17–23 years of age were surveyed. Other exclusion criteria included individuals with cognitive impairment or active mental illness, all incomplete responses, history of lower urinary tract surgery or cancer, neurogenic diseases affecting urination(as estimated by the students with instructions on how to fill this in in the instruction pamphlet).

### Statistical analysis

The data set was established using Epidata software (Version 3.1, Odense, Denmark). The Statistical Package for the Social Sciences (SPSS, Version 23.0 for Windows) was used for data analysis. Continuous variables were expressed as the mean ± standard deviation for normally distributed variables or as the median ± interquartile range for nonnormally distributed variables. The t-test or Mann–Whitney U test was used for comparisons between the two groups. Moreover, categorical variables were presented using frequencies or percentages and were assessed by the chi-square test. Linear regression was used to analyze the correlation of entrance exam scores. The independent predictors were further determined by multivariate logistic regression analysis. *P* < 0.05 was considered to indicate statistical significance.

## Results

### Prevalence of nocturia

A total of 13,874 questionnaires were collected and 13,104 (94.45%) qualified for statistical analysis. A total of 659 questionnaires (5.03%) indicated nocturia ≥ 2 times per night, 3618 (27.61%) indicated nocturia once per night and 8827 (67.36%) indicated no nocturia. The prevalence of CRN in different age groups was shown in Table 1.

**Table 1:** Comparison of prevalence rates in different age groups.

Age	Male		Female		Total		*P*-value	
	No	CRN	No	CRN	No	CRN		
17	175	6 (3.43)	243	10 (4.12)	418	16 (3.83)	0.130	0.718
18	1193	55 (4.61)	1788	97 (5.43)	2981	152 (5.10)	0.982	0.322
19	1545	61 (3.95)	2296	107 (4.66)	3841	168 (4.37)	1.120	0.290
20	1151	58 (5.04)	1786	95 (5.32)	2937	153 (5.21)	0.111	0.739
21	800	39 (4.86)	1167	70 (6.00)	1967	109 (5.54)	1.144	0.285
22	232	12 (5.17)	320	21 (6.56)	552	33 (5.98)	0.462	0.497
23	166	9 (5.42)	242	19 (7.85)	408	28 (6.86)	0.909	0.340
Total	5262	240 (4.56)	7842	419 (5.34)	13,104	659 (5.03)	4.032	0.045

CRN, clinically relevant nocturia.

### Relevant factors

The students in the CRN group and control group were comparable in average age being 20.03 ± 1.58 and 19.46 ± 1.33, respectively. Both univariate analysis and the logistic stepwise regression model showed that female, urinary frequency and urgency, RUTI, ease of waking up, and history of enuresis are relevant factors for nocturia occurrence in Tables 2 and 3.

**Table 2:** The prevalence and relevant factors for clinically relevant nocturia in Chinese university students (univariate analysis).

Relevant factor	No	CRN		t/χ^2^	*P*-value
		Yes	No		
Age	13,104	20.03 ± 1.58	19.46 ± 1.33	− 10.616	< 0.001
Gender					
Male	5262	240 (4.56)	5022 (95.44)	4.032	0.045
Female	7842	419 (5.34)	7423 (94.66)		
Place of residence					
Urban	5028	262 (5.21)	4766 (94.79)	0.933	0.334
Rural	8076	397 (4.92)	7679 (95.08)		
Sleep conditions					
Easy to wake up	4918	276 (5.61)	4642 (94.39)	5.603	0.018
Not easy to wake up	8186	383 (4.68)	7803 (95.32)		
History of enuresis					
Yes	249	22 (8.84)	227 (91.16)	7.700	0.006
No	12,855	637 (4.96)	12,218 (95.04)		
RUTI					
Yes	568	41 (7.22)	527 (92.78)	5.958	0.015
No	12,536	618 (4.93)	11,918 (95.07)		
Frequency					
Now	253	21 (8.30)	232 (91.70)	16.358	< 0.001
Past	748	56 (7.49)	692 (92.51)		
Never	12,103	582 (4.81)	11,521 (95.19)		
Urgency					
Now	157	15 (9.55)	142 (90.45)	11.871	0.003
Past	692	47 (6.79)	645 (93.21)		
Never	12,255	597 (4.87)	11,658 (95.13)		
DUI					
Now	117	7 (5.98)	110 (94.02)	2.114	0.347
Past	541	34 (6.28)	507 (93.72)		
Never	12,446	618 (4.97)	11,828 (95.03)		

Values are presented as number (%). UTI, urinary tract infection; DUI, Day time urinary incontinence; CRN, clinically relevant nocturia.

**Table 3:** Logistic regression analysis of relevant factors of clinically relevant nocturia prevalence.

	B	SE	Wald value	OR	95% CI	*P*-value
Male	− 0.165	0.083	3.946	0.8481	0.720–0.998	0.047
Frequency (now)	0.440	0.236	3.476	1.553	0.987–2.465	0.062
Frequency (past)	0.398	0.147	7.325	1.489	1.116–1.987	0.007
Urgency (now)	0.649	0.276	5.512	1.914	1.113–3.290	0.019
Urgency (past)	0.266	0.159	2.809	1.305	0.956–1.781	0.094
RUTI	0.282	0.171	2.710	1.326	0.948–1.856	0.100
History of enuresis	0.549	0.230	5.701	1.731	1.103–2.716	0.017
Sleep condition	0.168	0.082	4.190	1.183	1.007–1.390	0.041

RUTI, recurrent urinary tract infection; OR, odds ratio; CI, confidence interval.

### College entrance examination

Linear regression analysis was used to analyze the correlation between entrance examination scores and nocturia, gender, urgency, frequency, RUTI. There was a correlation between entrance examination scores and nocturia in Table 4 (*P* < 0.001). The T test was used to analyse the correlation between the number of episodes of nocturnal urination and university entrance examination scores. The subjects were divided into different groups by frequency of nocturnal urination. Entrance scores significantly decreased with an increase in the number of nocturia episodes per night in Table 5 (*P* < 0.001).

**Table 4:** Linear regression Analysis of entrance exam scores.

	B	SD	Beta	t	*P*-value
Const	478.548	4.847		98.733	< 0.001
No nocturia	18.807	1.813	0.090	10.370	< 0.001
Male	− 0.553	0.808	− 0.006	− 0.685	0.493
Frequency(never)	1.983	2.895	0.012	0.685	0.493
Frequency(past)	4.202	3.303	0.021	1.272	0.203
Urgency (never)	0.958	3.644	0.005	0.263	0.793
Urgency (past)	0.242	4.008	0.001	0.060	0.952
RUTI	3.454	1.962	0.015	1.760	0.078

SD, standard deviation; RUTI, recurrent urinary tract infection; r, correlation coefficient.

**Table 5:** The relationship between the number of nocturia episodes and the university entrance score.

Voids/night	College entrance examination		N
	Average	Standard Deviation	
0	507.14	44.79	4431
1	496.45	45.61	8014
2	482.64	40.17	604
3	471.89	22.61	47
≥ 4	456.25	33.33	8
Total	499.32	45.49	13,104

### Sleep quality

According to the T test, the total scores of sleep quality, sleep duration, sleep efficiency, sleep disturbance and daytime dysfunction in the CRN group were significantly higher than those in the control group (those with no nocturia). The scores of sleep latency and sleeping medication use in the CRN group were also higher than those in the control group, but no statistical difference was found in Table 6.

**Table 6:** The effects of CRN on sleep quality.

	Nocturia	No nocturia	t	*P*-value
C1 (sleep quality)	0.95 ± 0.96	0.81 ± 1.00	− 3.511	< 0.001
C2 (sleep latency)	1.28 ± 0.97	1.23 ± 0.88	− 1.391	0.164
C3 (sleep duration)	0.90 ± 0.89	0.65 ± 0.95	− 6.456	< 0.001
C4 (sleep efficiency)	0.79 ± 0.94	0.70 ± 0.90	2.702	0.007
C5 (sleep disturbance)	0.81 ± 0.99	0.65 ± 0.95	− 4.621	< 0.001
C6 (sleeping medication use)	0.28 ± 0.61	0.27 ± 0.57	− 0.450	0.653
C7 (daytime dysfunction)	1.69 ± 0.71	1.34 ± 0.80	− 11.237	< 0.001
PSQI score	6.71 ± 2.28	5.65 ± 2.27	− 11.640	< 0.001

CRN, clinically relevant nocturia; PSQI, pittsburgh sleep quality index.

## Discussion

The present study found that female gender, ease of waking up, urgency, frequency, RUTI and enuresis were associated relevant factors of nocturia. The Chinese university students with nocturia suffered from significantly more sleep problems which may have negative impact on learning.

Nocturia was described as the most bothersome of all urinary symptoms, which had a high prevalence and complex etiology. Some patients had insufficient understanding of nocturia, reflected in the reluctance to seek medical help. It had been reported that the prevalence of nocturia increases with age, and the condition had a serious impact on patients' physical health, sleep quality and quality of life^15^.

At present, the average age of patients in studies of nocturia was more than 30 years old^16^. Studies of the effects of nocturia on young adults were rare. Many researchers had focused on the prevalence and related factors in middle-aged and elderly people, but few had performed similar studies in young adults. Therefore, the present study explored the prevalence of nocturia in the young adult population and investigates its relevant factors and effects foe learning and sleep quality.

Wide variations in the estimated prevalence of nocturia were to be expected, largely due to the differences in nocturia definitions, specific population, and data collection method. A study of individuals over 40 years of age found that the prevalence of ≥ 2 nocturia episodes per night was recorded as 37.9% (men: 35.2%, women: 40.6%)^17^. When the age increases to 80, the prevalence of nocturia was as high as 60%. According to a study by Bliwise with a prevalence of 4.4%-18% in women in their second or third decaded and 28.3–61.5% in their seventh and eighth decades^18^. In men, the corresponding age groups demonstrated a prevalence of 2–16.6% and 29–59.3%, respectively. Similarly, an epidemiological survey of nocturia from Chinese mainland found that 57.5% of participants reported voiding once per night, and 24.7% of participants reported voiding twice or more per night. The above results showed that nocturia prevalence increases with age. Our study found that the prevalence of nocturia in college students was not only common (about 5%), but also showed a significant negative impact on sleep and academic performance. However, no statistical evidence provided to support an increase in nocturia with age (from 17 to 23 years) in present study.

According to numerous reports in the literature, when nocturia events occur ≥ 2 times/night, this significantly affected quality of life and sleep quality and might have more serious consequences^19^. People who urinated only once at night were considered to have nocturia by definition, but this was considered acceptable as these people seemed to show minimal problems. Therefore, this study aimed to look at those with more significant nocturia of ≥ 2 times per night and for the purpose of this study to address these as clinically relevant nocturia (CRN).

Nocturia was an impactful health issue at risk of being missed, especially in younger female patients. A study from Vienna^20^, in women, only 12.3% with one nocturia event per night reported it as “quite a problem” or “a serious problem”; this percentage increases to 39.4% for those with two incidents per night, to 42.9% for those with three incidents per night, and to 80.0% for those with at least four incidents per night. In men, the respective percentages were 6.6%, 25.0%, 51.6%, and 70.0%. Obviously, when nocturia events occur ≥ 2 times/night, this significantly affected quality of life and sleep quality and might have more serious consequences. This was also the reason of present study used the definition of nocturia as s waking to urinate ≥ 2 times per night.

Our study found that 5.03% of the respondents had CRN of two or more times per night, 27.61% had nocturia once per night, and 67.36% no nocturia. Through our investigation, we found that among the subjects who experienced nocturia once a night, nocturia was not considered a disease and the condition had no significant effect on their study and life. Among the nocturia subjects with two or more voids per night, 57% of the subjects thought the nocturia needed treatment and had an impact on their study and daytime status.

The reasons for nocturnal group are more likely to wake up easily in present study might due to the detrusor overactivity which produced strong arousal stimuli that affect the awakening area of the brain (ascending activation system); another contribute factor of mental stress and fearing of bedwetting at night are also possible. This is also supported by literatures from Rackley et al.^21^ who found tolterodine significantly reduced OAB-related nocturnal micturition compared to placebo, and from Breyer^22^ who reported a bidirectional association between depression and nocturia. It is indicated that screening and treatment bladder dysfunction and mental stress is very important in cases with clinical nocturia. However, the involved mechanism needs to be further investigated in the future.

Previous studies had explored the gender differences in nocturia, but mainly focused on middle-aged and elderly patients, while there were few studies on gender differences in young people. An epidemiological survey of Koreans^23^ found that the prevalence of nocturia was higher in women aged 40 to 59 than in men of the same age, while this prevalence was the same in men and women between the ages of 70 and 80 years. A prevalence survey of a large sample showed that the prevalence of nocturia was greater in women of ages 18–49 years than men of the same age group. Among individuals of ages18–29, the prevalence of nocturia in women was three times that in men. The gap in prevalence, however, closed gradually with aging. Among individuals of ages 30–39, the prevalence of nocturia in women was only 50% higher than in men; the prevalence was equal across sexes at 50–59 years. At older ages, the prevalence of nocturia in men could be higher than that in women. Moreover, a study associated delivery and lower urinary tract function^24^. Indeed, women with a history of delivery had a higher prevalence of nocturia than women without a history of delivery, and the incidence of nocturia would gradually increase with the increase of the number of deliveries. The authors also mentioned that the mode of delivery might also affect the occurrence of nocturia. Moreover, nocturia was a little more frequent among young women than young men, but equally or more common among men in the elderly, in accord with several studies across different countries and continents^20,25^. The explicit explanations for the gender difference remained unknown, but it was plausible that young women were likely to be vulnerable to greater fragmentation of sleep due to other reasons such as anxiety or insomnia, hence resulting in more voids at night. The prevalence of nocturia was the higher in men than women in elderly patients, which might be due to the fact that elderly men were prone to nocturia caused by prostate hypertrophy^26^.

Nocturia was a highly multifactorial condition. Causes of nocturia included urologic and non-urologic factors. Urologic causes of nocturia might include reduced bladder capacity, nocturnal polyuria, detrusor overactivity and mixed aetiology^27–29^. Non-urologic causes of nocturia also included different factors, such as higher BMI, smoking, drinking, hypertension and diabetes mellitus^30,31^. These factors were common in middle-aged and elderly people, and their influence became more pronounced with age. Therefore, the observed age-related increase in the prevalence of nocturia in middle-aged and elderly people might be related to these factors. However, the influence of these factors on young people had not been reported in the literature. Whether the causes of nocturia and the pathophysiological mechanisms involved in the young adult population were similar or different from those of the middle-aged or elderly population must be further researched.

Present study showed 1.9% of cases suffered from nocturnal enuresis^11^, which was similar to our previous study of 1.17% of prevalence, where the relationship between enuresis and nocturia was not mentioned although the frequency, urgency, RUTI had been indicated as relevant factors of enuresis. Nocturnal enuresis and nocturia were conditions in which the diurnal urination rhythm was disturbed. Nocturnal enuresis might represent an immature development of the rhythm whereas nocturia might reflect degeneration of the rhythm. The cross‐sectional analysis showed that a history of nocturnal enuresis was significantly correlated with nocturia^27^, and the ratio of participants with a medical history of enuresis was increased along with night‐time frequency. In the longitudinal analysis, a medical history of nocturnal enuresis had a significant relationship with the progression of nocturia, Fundamental causes of nocturnal enuresis and nocturia were not sufficiently understood to allow the pathogenic relationship to be determined. In our study, we analyzed the relationship between nocturia and history of nocturnal enuresis, LUTS and recurrent urinary tract infection, and found that a higher proportion of people with nocturia had a history of nocturnal enuresis, which further proved the correlation between history of nocturnal enuresis and the occurrence of nocturia. These results suggested that a history of nocturnal enuresis were independent relevant factors for nocturia.

Our study found that the incidence of frequent urination, urgency and RUTI increased significantly in young college students with clinically relevant nocturia. Therefore, we speculated that bladder dysfunction such as detrusor overactivity, small bladder capacity as well as detrusor spinincter dysnergia might cause the occurrence of clinically relevant nocturia. LUTS/BPE often led to a reduced functional bladder capacity, induced involuntary detrusor contractions, or high post-void residuals^27^, therefore increasing frequency of nocturnal voiding.

Sleep quality played a vital role in physical and mental functioning. A large number of observational studies demonstrate a relationship between the frequency of nocturnal voiding and the negative effect on QoL and well-being^32, 33^. Hence, we should focus on the relationship between nocturia and daytime QOL in the young university students. A large survey of nocturia related to sleep quality and daytime quality of life in a young Japanese population^34^ demonstrated that the first uninterrupted sleep period (FUSP), total sleep time (TST), sleep quality and daytime quality of life decreased with the increase in the number of nocturia at night. National Sleep Foundation survey in the USA in 2003 identified nocturia as the attributed cause of sleep disturbance every night or almost every night in 53% of those aged 55–84 years. Sleep disturbance due to nocturia was more than 4 times more prevalent than pain^35^. Polysomnographic data from the Sleep Heart Health Study demonstrated that patients with nocturia had less total sleep time, sleep efficiency and proportion of REM sleep, but had a higher arousal index and were more hypoxemic, the latter indicating a higher likelihood of sleep disordered breathing or apnoea^36^.

Similarly we could also demonstrate that the CRN group had poor sleep quality, shorter sleep time, poor sleep efficiency, obvious sleep disorders and obvious daytime dysfunction. As it required the individual to wake up and void at night, it might be a cause of sleep disturbance and poor daytime performance. We also found that people who were easily woken up from sleep had a higher prevalence of CRN. Therefore, we believed that the improvement of nocturia could prolong the total sleep time, improve the score of Pittsburgh Sleep Quality Index and improving the quality of daytime life.

In this study, we compared the university entrance examination scores of students with the number of nocturia episodes per night and found that students with increasing number of nocturia episodes achieved lower scores. This examination in China was a national unified examination organised and administered by the Chinese Ministry of Education, which occurred around two months before the time of university entrance. It was both the fairest and strictest test in China and thus can thoroughly reflect the students' learning achievements. With this finding and the finding of sleep disturbance amongst those with CRN, we postulated that CRN might be a cause for poor daytime cognitive function in young adults reflected in the poorer educational achievement. CRN and poor sleep might lead to drowsiness, fatigue, and inattention during the day. Although learning might be affected by many factors, nocturia could obviously be one of them. One limitation from our study was that the college entrance examination was taken before entry into University. We did not have data on the exact onset time of the nocturia and therefore did not know whether the score was a direct result of pre-existing nocturia or whether a poorer academic achiever had a higher chance of nocturia later on. A follow-up to this would be to have a longitudinal assessment of academic achievement or to have a follow-up questionnaire to assess the onset time of nocturia.

There were some limitations of this study. The respondents were not randomly selected for the survey, there was over representation of females and the respondents completed the questionnaire based on their memory, which mighty lead to recall bias, therefore, it might not be a complete representation of all young Chinese university students. Considering the nocturia once a night did not show a significant effect on daily life and study in the survey, we did not correlate these cases with those had clinically relevant nocturia. In addition, due to the small number of subgroup analysis, we did not conduct subgroup analysis. A multicentre, large sample and randomised study is needed in the future to address further issues of nocturia in young university students.

In summary, our study showed that the prevalence of nocturia in young adult was high and increasing with increasing age. Nocturia seemed related to gender of female, ease of waking up, urgency, frequency, RUTI and enuresis. In addition, we found that nocturia significantly affected sleep quality and possibly congnitive performance. Therefore, nocturia deserved more attention in this age group of young university students.

## Conclusion

Nocturia was common in Chinese university students. Female, ease of waking up, urgency, frequency, RUTI and enuresis were associated relevant factors of CRN. Moreover, young adults with CRN suffered from significantly more sleep problems including sleep disturbance than controls which might have negative impact on learning as suggested by lower achievements in their university entrance scores suggesting a need for more awareness and earlier active intervention for CRN.

## Data Availability

The data for the current study used for statistical analysis are available from the corresponding author upon reasonable justification.
